# Host Switching of Zoonotic Broad Fish Tapeworm (*Dibothriocephalus latus*) to Salmonids, Patagonia

**DOI:** 10.3201/eid2511.190792

**Published:** 2019-11

**Authors:** Roman Kuchta, Alžbeta Radačovská, Eva Bazsalovicsová, Gustavo Viozzi, Liliana Semenas, Marina Arbetman, Tomáš Scholz

**Affiliations:** Biology Centre of the Czech Academy of Sciences, České Budějovice, Czech Republic (R. Kuchta, T. Scholz);; Institute of Parasitology, Slovak Academy of Sciences, Košice, Slovak Republic (A. Radačovská, E. Bazsalovicsová); U; niversidad Nacional del Comahue, San Carlos de Bariloche, Argentina (G. Viozzi, L. Semenas, M. Arbetman)

**Keywords:** Diphyllobothrium, diphyllobothriosis, parasites, cestode, host switching, zoonotic infections, zoonoses, broad fish tapeworm, Patagonia, Chile, Argentina, South America, *Dibothriocephalus latus*

## Abstract

Diphyllobothriosis is a reemerging zoonotic disease because of global trade and increased popularity of eating raw fish. We present molecular evidence of host switching of a human-infecting broad fish tapeworm, *Dibothriocephalus latus*, and use of salmonids as intermediate or paratenic hosts and thus a source of human infection in South America.

Diphyllobothriosis is an emerging zoonotic disease caused by broad fish tapeworms. Except for the Pacific broad tapeworm (*Adenocephalus pacificus*), whose life cycle is completed in the sea, all species of the genus *Dibothriocephalus* (formerly in *Diphyllobothrium*) were limited to the freshwaters in the Northern Hemisphere (*1*). However, some of these tapeworms also were reported in the Southern Hemisphere, including South America, especially Patagonia, in the 20th century. Although the introduction routes of these human parasites remain unknown, their larvae (plerocercoids) have appeared in South America in nonnative but economically important salmonids, such as rainbow, brown, and brook trout (*2*,*3*).

Several cases of diphyllobothriosis have been reported from South America, and plerocercoids of tapeworms identified as *Dibothriocephalus latus* and *D. dendriticus* have been reported in fish (Appendix Table 1). However, species identification was based almost exclusively on morphologic characteristics. Considering general uniformity, intraspecific variability, and shortage of species-specific morphologic traits (especially in plerocercoids), all previous reports of *D. latus* and *D. dendriticus* tapeworms from South America need verification (*4*). Reports concerning the most commercially important species of salmonids being infected with *D. latus* tapeworms are especially dubious because this species most likely uses only freshwater percid, esocid, and gadid fish as its second intermediate hosts in the Northern Hemisphere (*1*,*4*).

Reliable identification of plerocercoids, which are the source of diphyllobothriosis, is crucial from the epidemiologic point of view because salmonids are of great economic value in South America as a food source for local populations, sport fishing, and exportation (*5*). We provide molecular evidence of second intermediate or paratenic host switching of human-infecting *D. latus* tapeworms in Patagonia, South America. 

We found a total of 44 plerocercoids in 3 salmonid species: from Lake Gutiérrez, Rio Negro, Argentina (October 2017), rainbow trout (*Oncorhynchus mykiss*), of which 2/7 fish examined were infected; brown trout (*Salmo trutta*), of which 3/4 were infected; and brook trout (*Salvelinus fontinalis*), of which 5/10 were infected; and from Lake Alicura, Neuquén, Argentina (April 2018), brown trout, of which 3/4 were infected. Most plerocercoids were encysted in the body cavity, mainly among the pyloric ceca, and only a few were free in the muscle. We selected, photographed, and sequenced the partial *cox*1 gene of 22 larvae in accordance with the procedure described by Wicht et al. (*6*). We also photodocumented morphologic vouchers (hologenophores) of sequenced specimens (Figure).

**Figure:**
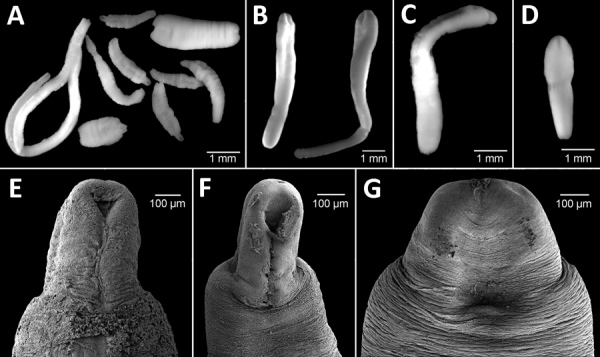
Microphotographs of *Dibothriocephalus* spp. plerocercoids from 3 salmonid species in Lago Gutiérrez, Patagonia, Argentina. A) *D. dendriticus* and *D. latus* plerocercoids from *Oncorhynchus mykiss* rainbow trout. B) *D. latus* plerocercoids from *Salvelinus fontinalis* brook trout. C) *D. dendriticus* plerocercoids from *Salmo trutta* brown trout. D) *D. latus* plerocercoids from *S. trutta* brown trout. E, F) *D. dendriticus* plerocercoids from *O. mykiss* rainbow trout. G) *D. latus* plerocercoids from *S. fontinalis* brook trout.

Our morphologic and molecular evaluation revealed the presence of *D. dendriticus* plerocercoids in 12 fish (8 in *O. mykiss* rainbow trout, 2 in *S. trutta* brown trout, and 2 in *S. fontinalis* brook trout); their sequences were identical with those of *D. dendriticus* tapeworms from Chile (GenBank accession nos. AB623150 and AB623149). We also detected the presence of *D. latus* plerocercoids in 10 fish (1 in *O. mykiss* rainbow trout, 3 in *S. trutta* brown trout, and 6 in *S. fontinalis* brook trout); these sequences were identical with those of *D. latus* tapeworms from Italy (GenBank accession no GU997614) (Appendix Table 2).

*D. dendriticus* plerocercoids have been reported in >50 species of freshwater fish of 12 families, and salmonids represent the principal, most common fish hosts (*7*). In contrast, *D. latus* plerocercoids have never been confirmed reliably in salmonids in the Northern Hemisphere, where they occur in relatively few freshwater fish species, such as perch (*Perca* spp.), pike (*Esox* spp.), ruffe (*Gymnocephalus cernua*), burbot (*Lota lota*), and walleye (*Sander* spp.) (*4*), which are not present in South America. Therefore, *D. latus* tapeworms had to adapt to new second or paratenic intermediate hosts (i.e., salmonids) after their introduction to the Southern Hemisphere, even though salmonids are not suitable hosts in the Northern Hemisphere, where these tapeworms occurred originally (*1*,*4*).

The origin of freshwater, human-infecting broad fish tapeworms in South America remains unknown. Salmonids were introduced to Chile (from Germany) and Argentina (from the United States) at the beginning of the 20th century as eggs or juveniles from a hatchery (*8*,*9*). However, no evidence indicates that naturally infected salmonids were imported to South America. Introduction of adult tapeworms of *Dibothriocephalus* spp. through infected humans or dogs cannot be ruled out, nor can the introduction of *D. dendriticus* tapeworms by migratory birds (*1*,*4*).

Our findings provide evidence of host switching of *D. latus* plerocercoids in Patagonia. Adaptation to new fish hosts might have serious epidemiologic consequences because of the economic importance of salmonids and their consumption by humans locally and abroad. Moreover, these introduced salmonids currently represent a substantial proportion of the total fish population in most of the lakes in the Andes region (*5*,*10*). Therefore, parasitologic examination of fish before their exportation on ice is necessary to avoid emergence of new foci of human diphyllobothriosis.

Appendix Additional information on host switching of zoonotic broad fish tapeworm (*Dibothriocephalus latus*) to salmonids, Patagonia.
