# Supplementary material for: Host Switching of Zoonotic Broad Fish Tapeworm (Dibothriocephalus latus) to Salmonids, Patagonia
Source: Emerg Infect Dis. 2019 Nov;25(11):2156–8. doi: 10.3201/eid2511.190792 (PMC6810218; doi:10.3201/eid2511.190792)
Supplement: Appendix — Additional information on host switching of zoonotic broad fish tapeworm (Dibothriocephalus latus) to salmonids, Patagonia. [file 19-0792-Techapp-s1.pdf]

Article DOI: DOI: https://doi.org/10.3201/eid2511.190792

Host-Switching of Zoonotic Broad Fish Tapeworm (*Dibothriocephalus latus*) to Salmonids, Patagonia

Appendix

Appendix Table 1. Second intermediate or paratenic hosts of *D. dendriticus* (*Dd*) and *D. latus* (*Dl*) reported from South America (Argentinean and Chilean Patagonia)*

Intermediate or paratenic hosts
1. *Basilichthys australis* (Atherinopsidae) – *Dd* (*2*), Chile; *Dl* (*3*), Chile
2. *Diplomystes camposensis* (Diplomystidae) – *Dd* (*4*), Chile
3. *Galaxias maculatus* (Galaxiidae) – *Dd* (*5*), Chile & *Dl* (*2*), Chile
4. *Galaxias platei* (Galaxiidae) – *Dl* (*2*), Chile
5. *Odontesthes mauleanum* (Atherinopsidae) – *Dd* & *Dl* (*3*), Chile
6. *Percichthys trucha* (Percichthyidae) – *Dd* (*5*), Chile & *Dl* (*2*), Chile
7. *Percilia gillissi* (Perciliidae) – *Dd* (*2*), Chile
8. **Oncorhynchus kisutch* (Salmonidae) – *Dd* (*6*), Chile
9. *Oncorhynchus mykiss (Salmonidae) – Dd (*7*, cited as D. microcordiceps), Argentina & Dl (*8*), Chile
10. **Salmo salar* (Salmonidae) – *Dd* & *Dl* (*7*), Argentina
11. **Salmo trutta* (Salmonidae) – *Dd* (*9*), Argentina
12. **Salvelinus fontinalis* (Salmonidae) *Dd* & *Dl* (*7*), Argentina

*Asterisks indicate introduced hosts. More recent data available at Rauque et al. (*1*).

Appendix Table 2. Sequenced samples of plerocercoids from Argentina. Results based on PCR amplification using species-specific primers for partial *cox*1 mt gene and consequent sequential analysis

Sample code	Locality	Host	Identification	GenBank no.
OM 1 A; RA-GU/1	Lago Gutiérrez	*O. mykiss*	*D. dendriticus*	MN326445
OM 1 B; RA-GU/2	Lago Gutiérrez	*O. mykiss*	*D. dendriticus*	MN326446
OM 1 C; RA-GU/3	Lago Gutiérrez	*O. mykiss*	*D. dendriticus*	MN326447
OM 1 C; RA-GU/4	Lago Gutiérrez	*O. mykiss*	*D. latus*	MN318155
OM 1 C; RA-GU/5	Lago Gutiérrez	*O. mykiss*	*D. dendriticus*	MN326448
OM 1 C; RA-GU/6	Lago Gutiérrez	*O. mykiss*	*D. dendriticus*	MN326449
OM 1 C; RA-GU/7	Lago Gutiérrez	*O. mykiss*	*D. dendriticus*	MN326450
OM 1 C; RA-GU/8	Lago Gutiérrez	*O. mykiss*	*D. dendriticus*	MN326451
OM 2 A; RA-GU/9	Lago Gutiérrez	*O. mykiss*	*D. dendriticus*	MN326452
TAM 4 A; RA-GU/10	Lago Gutiérrez	*S. trutta*	*D. latus*	MN318156
TAM 4 B; RA-GU/11	Lago Gutiérrez	*S. trutta*	*D. dendriticus*	MN326453
TAM 2; RA-GU/12	Lago Gutiérrez	*S. trutta*	*D. dendriticus*	MN326454
TAR 1 A; RA-GU/15	Lago Gutiérrez	*S. fontinalis*	*D. dendriticus*	MN326455
TAR 1 B; RA-GU/16	Lago Gutiérrez	*S. fontinalis*	*D. latus*	MN318157
TAR 2; RA-GU/17	Lago Gutiérrez	*S. fontinalis*	*D. latus*	MN318158
TAR 4 A; RA-GU/18	Lago Gutiérrez	*S. fontinalis*	*D. latus*	MN318159
TAR 4 B; RA-GU/19	Lago Gutiérrez	*S. fontinalis*	*D. latus*	MN318160
TAR 7; RA-GU/20	Lago Gutiérrez	*S. fontinalis*	*D. dendriticus*	MN326456
TAR 8 A; RA-GU/21	Lago Gutiérrez	*S. fontinalis*	*D. latus*	MN318161
TAR 8 B; RA-GU/22	Lago Gutiérrez	*S. fontinalis*	*D. latus*	MN318162
TAM 3 A; RA-AL/1	Lago Alicura	*S. trutta*	*D. latus*	MN318163
TAM 3 B; RA-AL/2	Lago Alicura	*S. trutta*	*D. latus*	MN318164

References

1. Rauque C, Viozzi G, Flores V, Vega R, Waicheim A, Salgado-Maldonado G. Helminth parasites of alien freshwater fishes in Patagonia (Argentina). Int J Parasitol Parasites Wildl. 2018;7:369–79. PubMed https://doi.org/10.1016/j.ijppaw.2018.09.008

2. Torres P, Franjola R, Pérez J, Auad S, Uherek F, Miranda JC, et al. Epidemiología de la difilobotriasis en la cuenca del Río Valdivia, Chile. Rev Saude Publica. 1989;23:45–57. PubMed https://doi.org/10.1590/S0034-89101989000100007

3. Torres P, Cuevas C, Tang M, Barra M, Franjola R, Navarrete N, et al. Introduced and native fishes as infection foci of *Diphyllobothrium* spp. in humans and dogs from two localities at Lake Panguipulli in Southern Chile. Comp Parasitol. 2004;71:111–7. https://doi.org/10.1654/4119

4. Torres P, Yera H. Diphyllobothriidae. 2018 [cited 2019 Sep 14]. https://www.waterpathogens.org/book/diphyllobothriidae

5. Torres P, Gesche W, Montefusco A, Miranda JC, Dietz P, Huijse R. Diphyllobothriosis humana y en peces del lago Riñihue, Chile: efecto de la actividad educativa, distribución estacional y relación con sexo, talla y dieta de los peces. Arch Med Vet. 1998;30:31–45. https://doi.org/10.4067/S0301-732X1998000100004

6. Torres P. Primeros registros de endohelmintos parásitos en el salmon coho, *Oncorhynchus kisutch* (Walbaum), introducidos en Chile. Arch Med Vet. 1990;22:105–7.

7. Szidat L, Soria MF. Difilobotriasis en nuestro pais. Sobre una nueva especie de *Sparganum*, parasita de salmones, y de *Diphyllobothrium*, parasita de graviotas, del Lago Nahuel Huapi. Boletín del Museo argentino de ciencias naturales. Bernardino Rivadavia. 1957;9:1–22.

8. Neghme A, Bertin V, Tagle I, Silva R, Artigas J. *Diphyllobothrium latum* en Chile. II. – Primera encuesta en el Lago Colico. Bol Chil Parasitol. 1950;5:16–7.

9. Revenga J, Acuic T, Semenas L. Difilobotriasis en salmónidos introducidos en el Parque y Reserva Nacional Nahuel Huapi Argentina: morfológia de plerocercoides. Arch Med Vet. 1991;23:157–63.
